# The Role of Podoplanin in the Biology of Differentiated Thyroid Cancers

**DOI:** 10.1371/journal.pone.0096541

**Published:** 2014-05-05

**Authors:** Magdalena Rudzińska, Damian Gaweł, Justyna Sikorska, Kamila M. Karpińska, Mirosław Kiedrowski, Tomasz Stępień, Magdalena Marchlewska, Barbara Czarnocka

**Affiliations:** 1 Department of Biochemistry and Molecular Biology, Center of Postgraduate Medical Education, Warsaw, Poland; 2 Department of Pathology, Maria Skłodowska-Curie Memorial Cancer Center and Institute of Oncology, Warsaw, Poland; 3 Department of General and Endocrinological Surgery, Copernicus Memorial Hospital, Łódź, Poland; Institut de Génomique Fonctionnelle de Lyon, France

## Abstract

Podoplanin (PDPN), a mucin-type transmembrane glycoprotein specific to the lymphatic system is expressed in a variety of human cancers, and is regarded as a factor promoting tumor progression. The purpose of this study was to elucidate the molecular role of PDPN in the biology of thyroid cancer cells. PDPN expression was evaluated in primary thyroid carcinomas and thyroid carcinoma cell lines by RT-qPCR, Western blotting, IF and IHC. To examine the role of podoplanin in determining a cell's malignant potential (cellular migration, invasion, proliferation, adhesion, motility, apoptosis), a thyroid cancer cell line with silenced PDPN expression was used. We observed that PDPN was solely expressed in the cancer cells of 40% of papillary thyroid carcinoma (PTC) tissues. Moreover, *PDPN* mRNA and protein were highly expressed in PTC-derived TPC1 and BcPAP cell lines but were not detected in follicular thyroid cancer derived cell lines. *PDPN* knock-down significantly decreased cellular invasion, and modestly reduced cell migration, while proliferation and adhesion were not affected. Our results demonstrate that PDPN mediates the invasive properties of cells derived from papillary thyroid carcinomas, suggesting that podoplanin might promote PTC progression.

## Introduction

Differentiated thyroid carcinoma (DTC) is the most common human endocrine malignancy. Papillary (PTC) and follicular (FTC) thyroid carcinomas are two major DTC variants, with the former representing the most common type (80% of all DTC cases) [1]. The molecular pathogenesis of thyroid cancer involves several molecular signaling pathways preferentially altered in PTC and FTC. PTCs carry oncogenic point mutations in *B-RAF* and *RAS*, and chromosomal rearrangements resulting in *RET* and *TRKA* chimeric genes, all of which may activate the mitogen-activated protein kinase (MAPK) pathway [2]. Activating *BRAF* V600E point mutation appears in, on average, 45%, whereas *RET/PTC* rearrangements and *RAS* mutations in 10–20% PTC cases [3], [4,]. In FTCs, in addition to *RAS* mutation, the *PAX8/PPARγ* rearrangement and deregulation of PI3K/ATK/PTEN (phosphatidylinositol-3-kinase/ATK/phosphatase and tensin homolog deleted on chromosome 10) signaling cascade are frequently detected, that are associated with the progression and dedifferentiation through activation of PI3K and AKT and inactivation or loss of *PTEN* suppressor gene expression [5], [6]. Although PTC is considered to be an indolent lesion with a favorable prognosis, the development of lymph node metastases affects up to 50% of PTC patients and the further development of distant metastases in some diagnosed cancers reduces survival rates [7], [8]. A common feature of tumor expansion is the dissemination of primary cancer cells, which can occur *via* a number of routes. Clinicopathological data have shown that papillary carcinomas are prone to metastasize to regional lymph nodes, suggesting that cells are spread *via* the lymphatic system [9], [10]. The molecular mechanisms and genetic factors involved in the dissemination of PTC cells, which determining the metastatic potential of papillary thyroid cancer, remain largely unknown.

Metastasis of cancer cells is a multi-step process and various cellular factors expressed in tumors may be involved. Several studies have highlighted the significance of lymphangiogenic factors in the progression of diverse tumors and a number of regulatory molecules participating in lymphangiogenesis have been identified [11], [12], [13]. One of the key lymphangiogenic molecules is podoplanin (PDPN). Human PDPN, also known as T1α -2, PA2.26, gp38 or aggrus, is a 38-kDa type I mucin-like transmembrane sialoglycoprotein composed of a highly O-glycosylated extracellular domain, a single membrane-spanning region and a short cytoplasmic tail [14]. In normal human tissues, podoplanin is expressed in kidney podocytes, skeletal muscle, heart, placenta, lung, and elsewhere [15], [16], [17]. PDPN is expressed in the lymphatic endothelial cells (LEC), but not in blood endothelial cells (BEC), and thus represents a specific marker of lymphatic endothelium and lymphangiogenesis [11]. Despite the specificity of its expression in lymphatic endothelium, PDPN has also been detected in various cancers [18], [19], [20]. The biological function of PDPN has not been fully defined, but the available data strongly suggest that it may play an important role as a mediator of tumor cell invasion [21]. The detailed mechanism underlying the spread of differentiated thyroid tumor cells and cancer progression, and especially the contribution of pro-lymphangiogenic molecules to this process is poorly understood. Hence, the aim of this study was to characterize the expression and function of podoplanin in thyroid tumors biology. PDPN expression was examined in primary tumors and in a panel of thyroid cancer cell lines derived from papillary (TPC1 and BcPAP) and follicular (FTC133 and CGTH-W-1) thyroid carcinomas. We also investigated the role of PDPN in regulating hallmarks of the malignant cell phenotype: proliferation, adhesion, survival rate, motility, migration and invasion. To determine the function of this transmembrane glycoprotein in the metastatic behavior of papillary cancer cells, we performed RT-qPCR, immunofluorescence and immunohistochemistry, as well as Western-blot analyses, and examined *PDPN* knock-down in cultured cells. The obtained data strongly suggest that PDPN can be considered a pro-metastatic factor affecting the spread of PTC.

## Materials and Methods

### Ethics Statement

The study was approved by the Ethical Committee of Human Studies at The Centre of Postgraduate Medical Education. Tissue samples were obtained with the permission of the respective Ethical Committees (at the Cancer Center and Institute of Oncology, at the Copernicus Memorial Hospital, and at the Centre of Postgraduate Medical Education). Written informed consent was obtained from all patients involved in this study. No industry gave support for this study.

### Thyroid tissue samples and cell lines

For gene expression experiments, fresh frozen tissues specimens of papillary thyroid carcinoma (T) and adjacent normal thyroid tissue from the contralateral lobe (NT) were collected at the Maria Skłodowska-Curie Memorial Cancer Center and Institute of Oncology, at the Department of General and Endocrinological Surgery, Copernicus Memorial Hospital (Warsaw and Łódź, Poland). For immunohistochemical (IHC) analysis, archived formalin-fixed, paraffin-embedded tissues (different from those used in RT-qPCR) from patients who had undergone total thyroidectomy were used.

The series of 173 archived tissues comprised: 112 papillary thyroid carcinomas (94 classical PTC with papillary or mixed papillary-follicular growth pattern and 18 follicular papillary carcinoma variants- FvPTC), 27 follicular thyroid carcinomas (FTC), 24 follicular adenomas (FA) and 10 normal thyroid tissues (NT). Tissue samples were obtained with the permission of the respective Ethical Committees (at the Cancer Center and Institute of Oncology, at the Copernicus Memorial Hospital, and at the Centre of Postgraduate Medical Education).

For functional studies we used thyroid carcinoma cell lines derived from papillary (TPC1 and BcPAP) and follicular (FTC133 and CGTH-W-1) thyroid carcinomas and SV40-immortalized human thyroid epithelial line Nthy-ori 3-1(hereafter referred to us as NTHY) to represent normal thyroid cells. Cell lines were obtained from the German Collection of Microorganisms and Cell Cultures (BcPAP and CGTH-W-1), and from the European Collection of Cell Cultures (FTC 133 and Nthy-ori 3-1). The TPC1 cell line [22] was kindly provided by Dr. M. Santoro (The University of Naples Federico II, Italy) [23]. The cells were cultured in complete RPMI-1640 medium supplemented with 10% (v/v) FBS (Roche, Switzerland), with the exception of FTC133 cells, which were grown in complete DMEM/F-12 supplemented with 10% FBS (Life Technologies, Invitrogen, USA). All cells were incubated at 37°C in a humidified 5% CO_2_ atmosphere.

### RNA isolation and real-time (RT)-qPCR

Total RNA was isolated from human thyroid specimens and thyroid cancer cell lines using an RNA Mini Kit (A&A Biotechnology, Poland) according to the recommended protocol, and the RNA integrity was verified by agarose gel electrophoresis. Total RNA (1 µg) was used for cDNA synthesis with a High Capacity cDNA Reverse Transcription Kit (Life Technologies, Applied Biosystems, USA). Expression of the human *PDPN* and *18S rRNA* genes was quantified by RT-qPCR using the cDNAs as template in reactions containing the double-stranded DNA-specific dye SYBR Green I and Maxima Fluorescein RT-qPCR Master Mix (Thermo Scientific, Canada), and specific oligonucleotide primers (listed below), as described previously [24].

*PDPN* (NM_006474)

Forward: 5′-CGAAGATGATGTGGTGACTC-3′

Reverse: 5′-CGATGCGAATGCCTGTTAC-3′

*18S rRNA* (NM_02255)

Forward: 5′-CCAGTAAGTGCGGGGTCATAAG-3′

Reverse: 5′-CCATCCAATCGGTAGTAGCG-3′.

Amplification, data acquisition and data analysis were performed using the iQ5 Real-Time PCR Detection System and software (Bio-Rad, USA).

### PDPN silencing with small interfering RNA (siRNA)

TPC1 cells were transfected with siRNA (final concentration 30 nM) targeting human *PDPN*, (siPDPN, 5′-CGAAGACCGCUAUAAGUCUTT-3′; Life Technologies, Ambion, USA) and a universal negative control siRNA (Sigma-Aldrich, USA) using Lipofectamine 2000 (Life Technologies, Invitrogen, USA) in Opti-MEM (Roche, Switzerland) medium, according to the recommended protocols. The efficiency of *PDPN* gene inhibition was evaluated 48 h after transfection by RT-qPCR, Western blotting and immunofluorescence. The experiment was repeated four times.

### Western blotting

Harvested cells were washed twice with ice-cold phosphate-buffered saline (PBS) and resuspended in 1% NP-40 lysis buffer (150 mM sodium chloride; 1.0% NP-40; 0.5% sodium deoxycholate; 0.1% SDS; 50 mM Tris, pH 8.0) supplemented with 1% protease inhibitor and 1% phosphatase inhibitor cocktails (Roche, Switzerland). Proteins in the cell lysates were quantified and samples of 30 µg were resolved on 8% SDS-PAGE gels and then electro-transferred onto nitrocellulose membranes (Bio-Rad, USA). The membranes were blocked by incubation with 5% non-fat milk in TBS (0.1% Tween-20) for 1 h at room temperature, then incubated overnight at 4°C with primary anti-podoplanin mouse monoclonal antibody (D2-40, diluted 1∶1000; AbD Serotec, USA). After intensive washing, the membranes were incubated with HRP-conjugated affinity-purified goat anti-mouse secondary antibody (Jackson ImmunoResearch Laboratories, USA). Signals from reactive bands were visualized by enhanced chemiluminescence detection (SuperSignal^®^ West Dura, Pierce Chemical, USA) as previously described [25]. As a loading control, the membranes were incubated with monoclonal anti-β-actin antibody (diluted 1∶5000; Sigma-Aldrich, USA) in an identical manner.

### Immunofluorescent staining

Cells grown on glass coverslips were fixed with ice-cold methanol, blocked with 2% goat serum/2% BSA, and then incubated with primary anti-podoplanin D2-40 mouse monoclonal antibody (1∶50; AbD Serotec, USA). After several washes, the cells were incubated with DyLight^TM^549-conjugated anti-mouse IgG (Jackson ImmunoResearch Laboratories, USA). The cells were counterstained with the nuclear dye DAPI and visualized with a fluorescence microscope (AxioObserver D1, Zeiss, Germany) using a 100x oil-immersion lens.

### Immunohistochemistry (IHC)

Immunohistochemical staining analysis was performed on 4-µm thick sections of archived formalin-fixed, paraffin-embedded thyroid tissues: PTC (112 cases), FTC (27 cases), FA (24 cases), and NT (10 cases). Sections were deparaffinised and heat-induced antigen retrieval was performed in target retrieval solution (TRS, pH 9.0; DAKO, Denmark), heating for 20 min in a pressure cooker. The slides were then incubated with anti-podoplanin D2-40 antibody for 1 h at room temperature. After washing, Envision+ antibody reagent was applied (DAKO, Denmark). Reactions were developed using diaminobenzidine (DAB) as the chromogenic substrate. The sections were counterstained with haematoxylin, mounted and examined under a light microscope. Negative controls were prepared following the same procedure but omitting the incubation with primary antibody. IHC staining was evaluated by two independent observers (MK and BC) and scored as “negative” or “positive,” according to the relative intensity of PDPN staining. The immunohistochemical data were subjected to statistical analysis.

### Cell migration and invasion assays

Cell migration and invasion activity were determined using a Boyden insert chamber (8-µm pore size, BD Falcon™ Cell Culture Inserts, USA) and BD BioCoat Matrigel Invasion Chamber 8-µm (BD Bioscience, USA), respectively. Cells were harvested and resuspended in serum-free medium, then counted with an EVA Automatic Cell counter (Nano EnTek, Korea). A total of 2×105 cells resuspended in RPMI 1640 medium (for both migration and invasion assays) were added to the chambers and incubated for 24 h at 37°C in a humidified 5% CO2 atmosphere. RPMI medium supplemented with 10% FBS was used as the chemoattractant. Cells that had migrated or invaded through the membrane were fixed and stained using a Diff-Quik Kit (Medion Diagnostics, Switzerland), imaged at 40x magnification with an Olympus BX41 microscope, and counted. Each experiment was performed in triplicate and repeated three times.

### *In vitro* wound healing motility assay

Scratches of comparable dimensions were made on confluent cell monolayers (grown in 6-well plates) using a sterile 200 µl pipette tip. The monolayers were then washed with PBS to remove detached cells and cell debris, and then refilled with growth medium and incubated at 37°C in a humidified 5% CO_2_ atmosphere. The plates were observed and photographed under a light microscope (10x, AxioObserver D1, Zeiss, and 20x Nikon Diaphot 300) every 3 h to determine the speed of wound closure. The width of the wounds at each time point was measured in 5 independent fields using ImageJ software (www.rsbweb.nih.gov). The cell migration distance was determined by measuring the width of the wound divided by two and by subtracting this value from the initial half-wound width [26]. Distance was quantified as follows: with the 10x lens, 1 pixel  = 1.026 µm; with the 20x lens, 1pixel  = 0.43 µm.

### Cell viability assay

To evaluate cell proliferation, the number of viable cells at 24 and 48 h after transfection with siRNA was determined using a 2,3-bis-(2-methoxy-4-nitro-5-sulfophenyl)-2H-tetrazolium-5-carboxanilide (XTT) tetrazolium cell proliferation kit (EZ4U, Biomedica GmBH, Austria). Briefly, wells of 96-well plates were seeded with 8×10^3^ cells (5 replicates). Then, 25 µl of XTT mixture reagent were added to each well and absorbance at the test wavelength (450 nm to measure formazan production) and the reference wavelength (620 nm) was measured at different time points using a Labsystems Multiscan RC microplate reader (Thermo Fisher Scientific, USA).

### Apoptosis assay

Apoptosis was evaluated using an Annexin V-FITC Apoptosis Detection Kit (Abcam, UK) following the recommended protocol. Briefly, harvested cells were washed with PBS, incubated with FITC-Annexin V and propidium iodide for 5 min at room temperature and then examined by flow cytometry (FACSCantoII, BD Biosciences, USA). The experiment was performed three times.

### Data analysis

All experiments were performed at least three times. Data are presented as the mean ±SEM. Statistical significance was determined using the nonparametric Mann-Whitney U test and paired t-test (GraphPad, Prizm, USA). A *P* value of <0.05 was considered statistically significant. Multivariate logistic regression model was used to examine the association between independent covariates and podoplanin tumorous expression.

## Results

### PDPN protein expression and cellular localization

**Figure 1:**
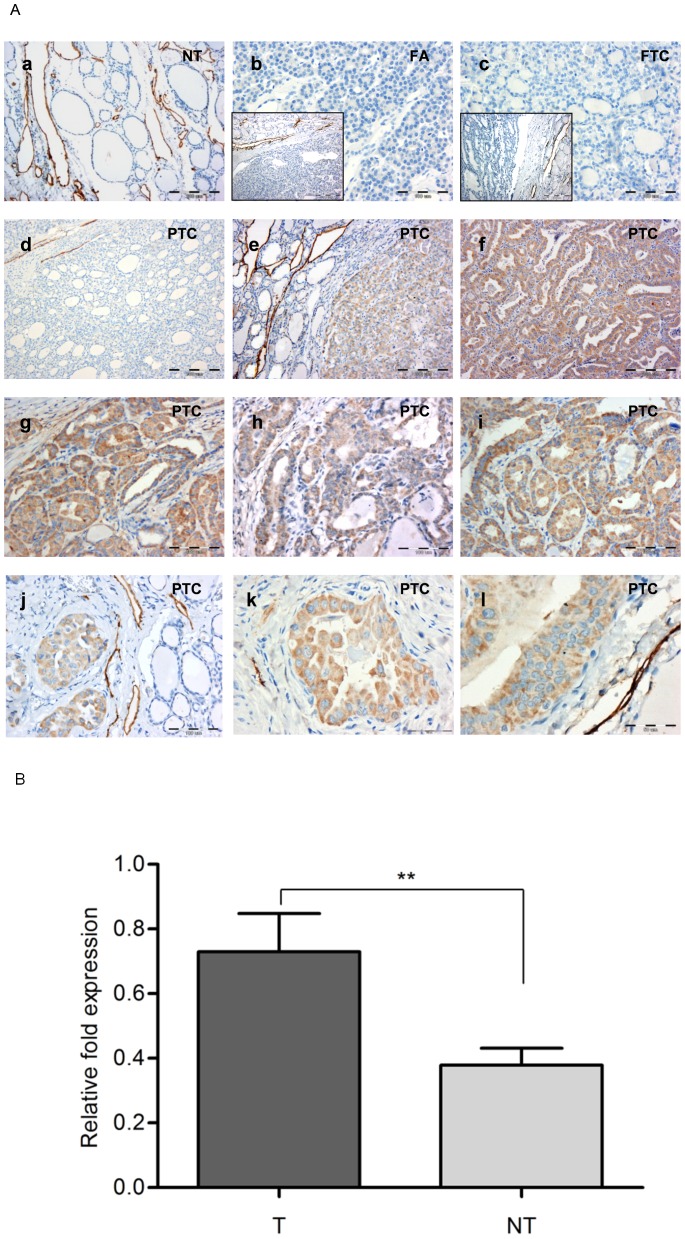
Podoplanin expression in human thyroid tissues analyzed by immunohistochemistry. A. Immunohistochemistry detection of podoplanin was carried on paraffin-embedded tissue sections. Representative immunostaining results obtained with anti-PDPN monoclonal antibody D2-40. (a) exclusive and strong staining of lymphatic vessels in normal thyroid tissue; (b) follicular adenoma (FA) tissue negative for podoplanin; (c) follicular thyroid carcinoma (FTC) tissue negative for podoplanin; (d) papillary thyroid carcinoma (PTC) case negative for podoplanin; (e-l) intense cytoplasmic staining of PDPN in PTC cells. In e, j and i intense staining of PDPN in tumor cells, with the peritumoral margin negative for podoplanin and strong staining of lymphatic vessels as an internal positive control. Original magnification: a-100x, a-inset -100x; b-200x, b-inset -100x; c-200x; d-200x, e-100x, f-100x, g-200x; h-200x, i-100x, j-200x, k-200x, l-400x. Of 112 PTC cases, 45 (40%) displayed ectopic podoplanin expression in the cancer cells. B. Relative PDPN mRNA expression in 21 PTCs (T) and paired normal tissues (NT) analyzed by RT-qPCR. PDPN and 18S rRNA transcript levels were quantified and podoplanin expression normalized against that of the housekeeping gene (18S rRNA). Results are presented as the mean ±SEM, **P<0.001.

The expression of podoplanin was first evaluated in archived thyroid tumor tissue samples. Immunohistochemical analysis was performed on a series of 173 paraffin-embedded tissues (112 PTC, 27 FTC, 24 FA and 10 NT) using anti-podoplanin monoclonal antibody D2-40. In normal thyroid tissue PDPN staining was absent in follicular cells and anti-PDPN antibody labeled solely and strongly only lymphatic endothelial cells in the numerous lymphatic vessels, which served as a good internal control (Figure 1A; a). All analyzed FTC and FA samples were negative for PDPN immunoreactivity (Fig. 1A; b, c). Majority of the examined PTCs were also negative for PDPN labeling (Fig. 1A; d). However, 40% (45 of 112) of PTCs showed positive immunostaining for podoplanin (Fig. 1A; e-l). Various intensity of PDPN labeling (from moderate to strong) was observed in the cytoplasm of tumor cells and in the most of cases, staining was distributed uniformly across the cancer. In contrast, peritumoral tissue cells (considered as “normal thyroid tissue”) were entirely PDPN negative (see Fig. 1A; e, j, k, l). In these tumor-free normal thyroid tissue margins, D2-40 antibody labeled exclusively and strongly the lymphatic vessels of different sizes and shapes. The association of PDPN expression in tumor tissue specimens with clinicopathological data was assessed by multivariate logistic regression model and summarized in Table 1. Cytoplasmic PDPN expression was not statistically significantly different between patients with larger or smaller tumor size (pT) or histological (FvPTC *vs* classic PTC) subtype. Although there was a highest number of cases (9 positive tumors among 17 tested) with induced podoplanin cytoplasmic expression in the pT3 group and the intensity of the staining was strong in all positive tumors, the number of cases in the group was too small to be statistically analyzed. However, the podoplanin protein expression was significantly correlated with the patient's age. Older PTC patients (≥45) showed more frequently (69% *vs* 31%) PDPN neoexpression then younger ones (<45), (adjusted odds ratio 4, 95% confidence interval 1.76–9.2, *P*<0.001).

**Table 1:** Correlation of podoplanin cellular expression with clinical and pathological parameters of human papillary carcinoma of the thyroid.

Variable	Number of cases	Podoplanin expression n (%)	*P* value
Age: 45.8±14.3; range 21–83	112	45 (40)	
Median 45.5			
**Gender**			
Male	15		
Female	97		
**Age at diagnosis**			
<45	52	14/45 (31.1)	*NS*
≥45	60	31/45 (68.9)	*0.001*
**Histological type**			
Classic PTC	92	39/45 (86.7)	
FvPTC	20	6/45 (13.3)	
FvPTC *vs* PTC		6/39	*0.524*
**pT-stage**			
pT1a	28	11/45 (24.4)	
pT1b	24	11/45 (24.4)	*0.648*
pT2	43	14/45 (31.1)	*0.866*
pT3	17	9/45 (20.1)	*0.275*

Abbreviation: NS =  non significant, (*P* value >*0,05*).

Our analysis was expanded by an investigation of *PDPN* gene expression in series of surgically removed PTC/NT paired tissues using RT-qPCR. In 14 of the 21 analyzed PTC/NT cases, a significantly higher (*P*<0.001) level of *PDPN* transcript was detected in PTC samples compared with the corresponding normal thyroid tissues (Fig. 1B). In the remaining 7 PTC/NT pairs, the level of *PDPN* mRNA in the PTC tissues was equal to or even lower than that in the paired normal tissues.

### Expression and cellular localization of PDPN in thyroid cancer cell lines

**Figure 2:**
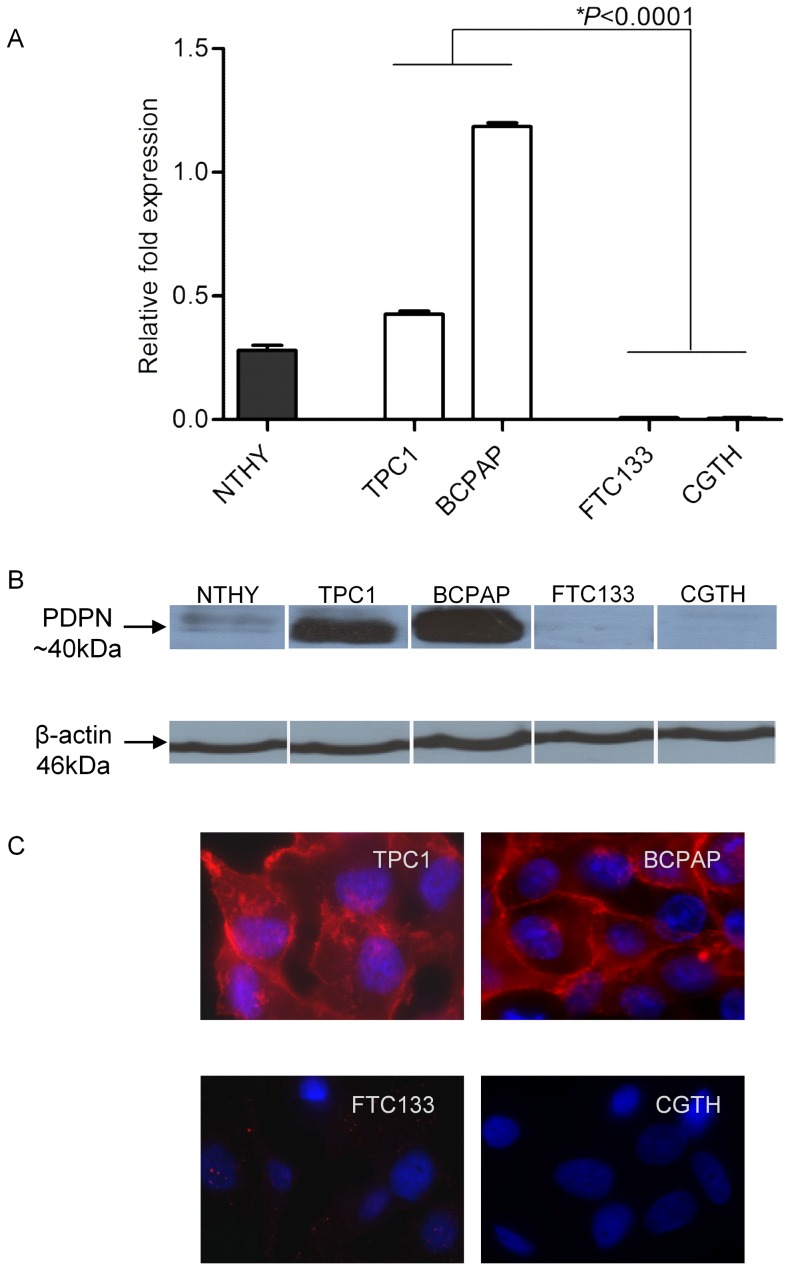
Podoplanin transcript and protein expression levels in differentiated thyroid cancer derived cell lines. A. PDPN mRNA expression in human thyroid cancer cell lines. RT-qPCR was used to evaluate levels of the transcript encoding podoplanin in total RNA prepared from NTHY control cells, PTC-derived TPC1 and BcPAP cell lines, and FTC-derived FTC133 and CGTH-W-1 cell lines. Data from four independent experiments performed in triplicate are expressed as the mean ±SEM, **P<0.001. B. Podoplanin protein levels in human thyroid cancer cell lines. Protein extracts (30 µg) were subjected to Western blot analysis with anti-PDPN monoclonal antibody D2-40 and β-actin antibody as a loading control. C. Immunofluorescence analysis of the podoplanin expression in TPC1, BcPAP, FTC133 and CGTH-W-1 thyroid cancer cell lines. PDPN was visualized by staining using anti-PDPN monoclonal antibody D2-40 followed by DyLight549-conjugated secondary antibody (red), and nuclei were counterstained with DAPI (blue). Magnification 1000x.

To investigate the functional role of podoplanin in thyroid cell biology we examined a normal thyroid cell line (NTHY), and a panel of papillary (TPC1 and BcPAP) and follicular (FTC133 and CGTH-W-1) thyroid cancer-derived cell lines. The level of *PDPN* mRNA was very low in the immortalized NTHY cells (Fig. 2A). In the thyroid carcinoma cells, remarkable differences in the relative *PDPN* transcript level between cells originating from PTC and FTC were observed. TPC1 and BcPAP cells exhibited the highest level of *PDPN* mRNA expression among all the tested thyroid carcinoma cell lines. In contrast, the *PDPN* transcript was not detected in the FTC133 and CGTH-W-1 follicular carcinoma-derived cell lines (Fig. 2A). On average, the level of *PDPN* mRNA in the PTC cell lines was more than 3-fold higher than that in the NTHY control cells.

### Podoplanin protein expression and cellular localization in thyroid cancer cell lines

To corroborate the RT-qPCR results, we carried out Western blotting and immunfluorescence analyses to evaluate PDPN protein expression and determine its cellular localization in thyroid cancer cell lines. The podoplanin protein levels estimated by immunoblotting were fully consistent with the RT-qPCR results. PDPN protein was strongly induced in TPC1 and BcPAP cell lines, but it was not detected in FTC-originating cells (Fig. 2B). Immunofluorescence analysis confirmed the high levels of PDPN in TPC1 and BcPAP cells. The protein was localized mainly at the cell membranes and in the cytoplasmic compartment of PTC-originating cell lines (Fig. 2C). The FTC133 and CGTH-W-1 lines were negative for PDPN immunostaining, again validating the RT-qPCR data.

### Effect of PDPN on the malignant cell phenotype

**Figure 3:**
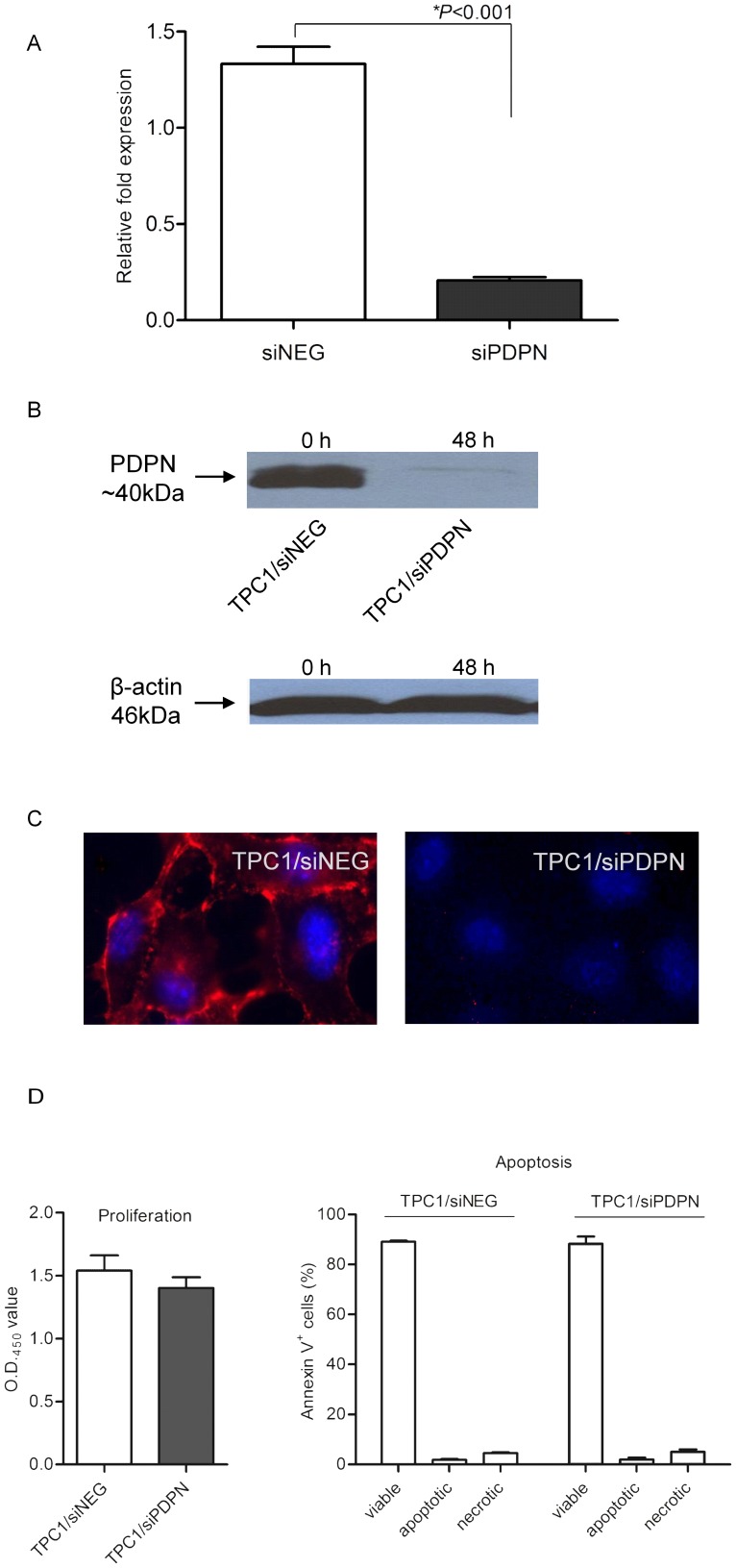
Down-regulation of podoplanin expression in TPC1 cells following transfection with PDPN-specific siRNA. A. RT-qPCR analysis of PDPN mRNA levels in TPC1 thyroid cancer cells 48 h after transfection with 30 nM siRNA specific for PDPN (siPDPN) or a negative control siRNA (siNEG). The results were normalized to the 18S rRNA level and bars represent the average fold change in PDPN transcript abundance in cells transfected with siPDPN compared with cells transfected with siNEG. The results are representative of four independent experiments. Data are presented as the mean ±SEM, **P<0.001. B. Western blot analysis of podoplanin and β-actin proteins in TPC1 cells before (0 h) and 48 h after transfection with siPDPN and control siNEG. C. Immunofluorescence staining of podoplanin protein in TPC1 cells transfected with siPDPN and control siNEG. Cells were stained with anti-PDPN monoclonal antibody D2-40 followed by DyLight549-conjugated secondary antibody (red), and counterstained with DAPI (blue). Magnification 1000x. D. Effect of PDPN on cell viability. D, left panel. Proliferation was measured at 24 and 48 h after transfection of TPC1 cells with siPDPN or control siNEG. Cells seeded in 96-well plates were treated with XTT mixture reagent and formazan formation was measured at 450 nm to determine the number of viable cells. Data are expressed as the mean ±SEM of at least three independent experiments performed in quintuplicate. D, right panel. Apoptosis was measured at 48 h after transfection of TPC1 cells with siPDPN or control siNEG. Cells were collected and stained with FITC Annexin V and propidium iodide, followed by flow cytometry. Representative measurements of the percentage of Annexin V+ cells are presented. Each bar represents the mean ±SEM of at least three independent experiments performed in quadruplicate. E. Function of podoplanin in cell adhesion. TPC1 cells transfected with siPDPN display adhesion capacity comparable to those of siNEG-transfected cells. Briefly, 8000 transfected cells were seeded in the wells of 96-well plates. After incubation for 24 or 48 h, the cell monolayers were washed, fixed with 4% formaldehyde for 15 min and stained with crystal violet (Merck, USA). The stained cells were lysed by treatment with 2% SDS, then the intensity of the released stain was quantified by spectrophotometry at 550 nm using a Labsystems Multiscan RC microplate reader (Thermo Fisher Scientific, Canada). Data represents three separate experiments.

In light of its pro-invasive properties reported in other types of human tumors and the high levels detected in PTC samples, we next investigated whether PDPN is involved in the migration and invasion of thyroid tumor cells. TPC1 cells were transfected with siRNA targeting *PDPN* (TPC1/siPDPN) and with a control universal negative siRNA (TPC1/siNEG). Any subsequent down-regulation of podoplanin was evaluated using RT-qPCR, Western blotting and immunofluorescence methods. Only ∼15% of the initial *PDPN* transcript level was detected in TPC1/siPDPN cells 48 h after transfection, confirming that the specific siRNA produced efficient silencing of *PDPN* (Fig. 3A). Western blotting and immunfluorescence assays demonstrated the knock-down of podoplanin gene expression in these cells. As shown in Fig. 3B and 3C, the PDPN protein was detected only in TPC1 cells transfected with the negative control siRNA. Cell proliferation, adhesion and survival were then assessed for the TPC1 cells transfected with the *PDPN*-specific and control siRNAs. No increase in the proliferation rate of cells with silenced *PDPN* was found compared to the control cells (Fig. 3D, left panel). We also did not observe any differences in the adhesion properties of *PDPN*-silenced and control cells (Fig.3E). Furthermore, there were no differences in the number of viable (>90%), apoptotic and necrotic cells between specific and non-specific siRNA-treated TPC1 cells (Fig. 3D, right panel), suggesting that the expression of PDPN does not increase the susceptibility of these cells to apoptosis.

### Migration and invasion

**Figure 4:**
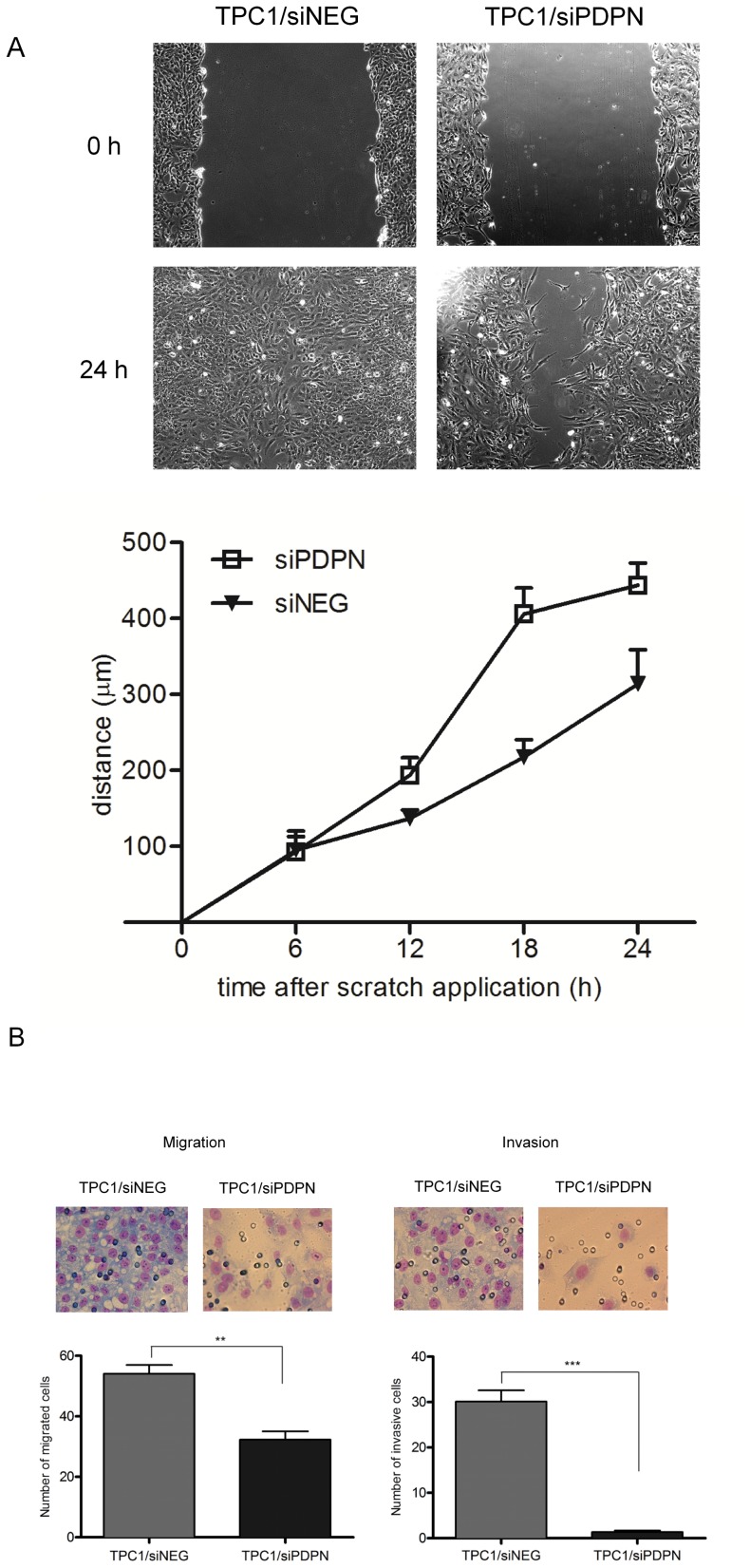
Silencing PDPN affects motility, migration and invasion ability of TPC1 cells. A. Scratch wound healing assay. Representative light microscope images showing healing of wounds in monolayers of TPC1 cells transfected with siPDPN or control siNEG, at 0 and 24% FBS as a chemoattractant and after 24 h, cells that had migrated through the 8-µm pore size membrane were stained and photographed at 40x magnification. The proportion of migrating cells is presented in graph form. Data are presented as the mean ±SEM of at least three separate experiments, **P<0.001. B, right panel. The invasiveness of TPC1 cells transfected with siPDPN or control siNEG was analyzed by adding cells to a BD BioCoat Matrigel Invasion Chamber, 8-µm. The lower reservoir was filled with 10% FBS as a chemoattractant and after 24 h, cells that had moved through the Matrigel to the lower surface of the membrane were fixed, stained and the photographed at 40x magnification. The proportion of invasive cells is presented in graph form. Data are presented as the mean ±SEM of at least three separate experiments, ***P<0.0001.

To analyze how reduced PDPN expression affects the migrative potential of TPC1 cells, we performed an *in vitro* scratch wound healing assay with cells transfected with siPDPN or with control siNEG. A scratch was made in monolayers of seeded cells and any differences in wound healing were monitored. In three independent experiments, healing by increased cell motility was found to be more rapid for control cells than for the *PDPN*-silenced cells (Fig. 4A). The motility of transfected cells was then assessed using a chamber migration assay. As expected, the migration of cells with reduced *PDPN* was clearly altered (Fig. 4B, left panel), being on average 2-fold lower than that of control cells. Then, we investigated the effect of *PDPN* knock-down on the invasive potential of the cells using the Matrigel invasion assay. Silencing of the *PDPN* gene profoundly impaired the invasive capacity of TPC1 cells (Fig. 4B, right panel). In each performed experimental replicate, we observed a consistent and strong reduction in the invasiveness of the *PDPN*-silenced cells as compared with the controls or the parental TPC1 cells (data not shown).

Taken together, these data demonstrate that podoplanin acts as a proinvasive factor in papillary thyroid carcinoma biology.

## Discussion

Although the earliest feature of disseminated disease in thyroid carcinoma is regional lymph node involvement little is known about the mechanisms by which cancer cells interact with lymphatic endothelial cells and then enter the lymphatic system. In particular, there is a lack of studies describing genetic factors involved in the metastatic process. Here we examined the potential role of podoplanin, a lymphangiogenic factor, in regulating the spread of thyroid cancer cells. Podoplanin is expressed in several normal cells and tissues and since it is selectively expressed in lymphatic endothelial cells (LEC) of lymphatic vascular system, it is used as a specific immunohistochemical marker distinguishing lymphatic and blood vessels. Moreover, several studies have identified PDPN as an important factor in the progression of several human tumors [27], [28], [29].

An increased level of PDPN was reported in highly metastatic clones of mouse colon adenocarcinoma and melanoma cell lines and in approximately 80% of human squamous cell carcinomas of the lung, larynx, cervix, skin, and oesophagus [21], [30], [31]. Moreover, Cueni *et al.* [32] identified an association between enhanced podoplanin expression, greater cell motility and increased tumor lymphangiogenesis and metastasis in the human MCF7 breast carcinoma xenograft model. They also showed a correlation between PDPN expression and a poor prognosis in patients with oral squamous cell carcinoma (OSCC).

To examine the role of podoplanin in thyroid cancer biology we investigated the expression profile of the PDPN transcript and protein in a series of DTC tissues and in papillary and follicular DTC-derived cell lines. We also examined the role of PDPN in promoting motility, migration and invasiveness of thyroid carcinoma cells.

Our experiments revealed that podoplanin is highly expressed in the neoplastic cells of many papillary thyroid tumors. The PDPN expression was detected in ∼40% of analyzed PTC cases and was absent in normal thyroid tissues and the peritumoral margin of “normal” unaffected tissues. In the PDPN-negative tissues, immunostaining was confined to the lymphatic vessels, which acted as a specific internal control. Since, the most common genetic alternation identified in PTCs is a somatic point mutation in the *BRAF* gene leading to a V600E substitution, we presumed that the *BRAF* V600E-activated constitutive signaling along the RET–RAS– BRAF–MAPK pathway may also affect podoplanin expression in PTCs. However, the immunohistological analysis of *BRAF*V600E in our series of archival tissues has not confirmed the effect of *BRAF* mutation on PDPN expression, as only ∼50% of PDPN positive cases were *BRAF* V600E positive. This suggests that the other signaling pathways, independent of *BRAF* mutation status, contribute to podoplanin neoexpression in PTCs. Surprisingly, in contrast to papillary thyroid carcinomas, all of the examined follicular thyroid carcinoma and follicular adenoma cases were negative for PDPN staining. Our observation corresponded with the previous studies, showing by IHC method that in the majority of PTC cases PDPN is expressed in tumor cells, whereas FTC, FA and normal thyroid did not express podoplanin [33]. This observation suggests that podoplanin is exclusively expressed in some PTC cases and may play an essential role in the biology of papillary tumors. These IHC findings were confirmed by the analysis of *PDPN* transcript levels in the frozen tissues of 21 PTC cases. The expression of *PDPN* mRNA was up-regulated in ∼70% of the analyzed tumor samples (by at least 2-fold) compared to paired normal thyroid tissues. The *PDPN* transcript levels in PTCs with *BRAF* V600E mutation were not statistically significantly different (*P*>0.05, data not shown) from the levels detected in PTC cases without *BRAF* mutation. This suggests again, that podoplanin expression in analyzed tumors is probably not affected by *BRAF* V600E mutation. The number of examined tissues, however, was low, and our data should be validated on larger number of *BRAF* V600E positive PTC cases. Podoplanin mRNA overexpression, in comparison to normal tissues, was also observed in some tumor tissues including colorectal cancer, oral squamous cell carcinoma or bladder cancer [34], [35], [36]. Moreover, a difference in the relative *PDPN* transcript level between the group of lymph node-negative or lymph node-positive cancer samples was also observed, suggesting the association between podoplanin expression and nodal metastasis or, as recently demonstrated, with distant metastases [35], [36]. Our findings cannot directly link the PDPN expression in PTC tumor cells with metastatic tendency in papillary cancer, mainly due to the lack of information regarding lymph node involvement. Podoplanin neoexpression in PTC cases was not related to the tumor size or conventional *vs* FvPTC histological/morphological tumor subtype. Nevertheless, among the PTCs examined in the present study, there were no tall cell variant of papillary carcinomas, which are considered as mostly associated with aggressiveness of PTCs [37]. We did not observe any statistically significant correlation in podoplanin expression between the samples from different tumor stages. Although the pT3 stage tumors showed some clear increase in PDPN protein expression (the staining was strong in all positive tumors), comparing to pT1 samples (9/17 *vs* 11/28; see Tab. 1), the number of available cases was not sufficient for statistical evaluation. Thus, we cannot rule out the possibility, that when the analysis will be performed on a larger group of PTCs the association between tumor size and podoplanin expression, or tall cell variant of PTC and PDPN positivity, will be found. Numerous clinical studies on factors influencing the PTC progression pointed out the significance of patient age [38], [39]. Lymph node metastases are regarded as prognostic factor of PTC patient's survival. There are some reports demonstrating higher frequency of nodal involvement in younger patients, whereas other point out the nodal involvement and patient's age ≥45 as factors affecting survival in patients with papillary thyroid carcinoma [40], [41]. We found that PDPN neoexpression is strongly correlated with older (≥45) patients age, which is one of the several classical clinico-pathological high-risk factors in papillary thyroid carcinomas. Taking into account that lymph node metastases in patients aged ≥45 correlates with unfavorable prognosis, our observation of high frequency of PDPN expression in tumor samples of these patients may imply the potential role of podoplanin in metastatic tendency of papillary thyroid carcinoma. Although the function of PDPN in tumor biology, including cancer progression, is not yet clear our data might link podoplanin expression with metastatic potential of PTCs.

We next examined the expression and function of podoplanin in DTC-derived cell lines. Podoplanin expression was significantly up-regulated in the papillary cancer-derived TPC1 (*RET/PTC1* rearrangement) and BcPAP (*BRAF* V600E mutation) cell lines, where BcPAP cells demonstrated higher *PDPN* mRNA expression than TPC1 cells, suggesting that more efficient activation of *PDPN* expression could be related to *BRAF* V600E gain-of-function mutation. Although, both oncoproteins, *BRAF* V600E and *RET/PTC1*, share common property of signaling *via* activation of MEK-ERK kinase pathway, they have unique phenotypic features, signifying that the different tumor biology may characterize cancers arising from different oncogenes [42], [43], [44]. In contrast, the follicular carcinoma derived FTC-133 cell line, carrying mutated tumor suppressor gene *PTEN*, and CGTH-W-1 cell line do not show the *PDPN* expression. The mechanism of *PDPN* gene down-regulation in FTC derived cells is unknown. One of the reasons could be, at least in the case of FTC-133 cells, the over-activation of PI3K/ATK signaling cascade due to the presence of the inactive *PTEN*. The tumor suppressor gene *PTEN* has been shown to play an important role in the pathogenesis of variety of human cancers including, the thyroid cancer [45], [46], [47]. The mechanism/s of *PDPN* down regulation in CGTH-W-1 follicular cancer cells is unknown, however, taking into account the diversity of the mechanisms by which the constitutive activation of PI3K/ATK signaling occur in cancer, its over-activation, among other signaling pathways, may also be considered.

The PDPN expression patterns in DTC-derived cell lines are similar to those observed in human PTC and FTC tissues. These data indicate that (i) podoplanin may play an important role in PTC cell biology, (ii) the mechanisms of progression of papillary and follicular carcinomas are likely to be different, and (iii) different sets of genetic factors may be required for the progression of PTCs and FTCs.

The process of carcinogenesis is comprised of multiple steps that correspond to different genetic alterations. The observed differences in PDPN expression in thyroid cancer cells might be due to the presence of known genetic alterations in PTC cells. Mutations in the *RET*/*RAS*/*B-RAF* genes of mitogen-activated protein kinase (MAPK) signal transduction pathways have been detected in the majority of DTCs [48]. These evolutionarily-conserved proteins control major cellular processes such as proliferation, differentiation, migration and apoptosis. The mutually exclusive *B-RAF V600E* mutation and *RET*/*PTC* rearrangements are most prevalent in papillary thyroid carcinomas (∼70% of all PTCs), and appear to be involved in the neoplastic transformation of follicular thyroid cells [4], [49]. The BcPAP and TPC1 cell lines, derived from PTCs, carry either of these genetic modifications (*BRAF* V600E and *RTE/PTC1* respectively) and both exclusively express podoplanin. Recently published data, with induced expression of *BRAF* V600E or *RET/PTC*, suggest that there are differences in the oncogenic strength and the molecular events, as well as differences in the genes affected by these two genetic changes [44], [50], [51], [52]. Therefore, taking into account that BcPAP line is derived, in fact, from poorly differentiated cancer, and has stronger oncogenic potential, which can not only initiate development of papillary tumors, but is also required to maintain and promote their progression, we choose for our study the TPC1 cell line, which is derived from well differentiated conventional PTC [53], [54].

Tumor cell motility, migration and invasion are necessary for metastasis. Although some data indicate that the expression of podoplanin in tumor cells might be related to their malignant potential, the functional contribution of this protein to cancer progression, invasion and metastasis remains unclear. To investigate the potential role of podoplanin in PTC metastatic activities, we examined its function in the regulation of the classical hallmarks of malignancy: proliferation, motility, migration and invasion. TPC1 cells deficient in *PDPN* expression were created by transfection with small interfering RNA (siRNA). We did not observe any clear differences in the proliferation, viability or adhesion of cells with and without PDPN expression. However, the *PDPN* knock-down reduced motility and migration, and dramatically changed the invasive properties of TPC1/si*PDPN* cells. We found, that *in vitro* silencing of PDPN expression leads to moderately reduced migration, as shown either in transwell migration assay or wound-healing assay, and caused profoundly reduced invasiveness of TPC1 cells, suggesting a potential role of this gene in the spreading of papillary thyroid carcinoma cells. Our observation agrees with several studies which demonstrated involvement of podoplanin in cell migration and invasiveness during cancer progression, and in the promotion of epithelial-mesenchymal transition through down-regulation of epithelial genes and up-regulation of mesenychymal markers [16], [55], [56]. The strong inhibition of the invasiveness of *PDPN*-silenced TPC1 cells implies an essential function for podoplanin in papillary cancer cell dissemination. Although the role of podoplanin remains poorly understood and the pathways involved in the pro-invasive phenotype of papillary thyroid cancer cells appear to be *multifactorial* and are largely uncharacterized, this is the first complex report describing the pro-metastatic activity of PDPN in papillary thyroid carcinoma biology.

The PDPN function we reveal is consistent with the majority of previous reports linking increased expression of podoplanin with tumor progression [21]. Several studies have shown that the presence of podoplanin is associated with lymph node metastasis of cancer cells and with poor prognosis. PDPN overexpression was identified as a pro-metaststic factor in squamous cell carcinoma [16], [32], larynx and oesophagus tumors [57], [58], and tumors of the central nervous system [59]. Other studies concerning astrocytic and gastric carcinomas, lung squamous cancer and other malignancies, have demonstrated the involvement of podoplanin in cell migratory activity necessary for metastasis [18], [60], [61]. Furthermore, it has been proposed that podoplanin might serve as a potential clinical marker for the malignant progression of oral leukoplakia [62], and it is regarded as a novel myoepithelial marker in salivary gland tumors [63].

The biological function of podoplanin seems to be complex. Our evidence of the pro-invasive character of high PDPN expression contrasts with reports showing that low expression of PDPN correlates with poor prognosis, e.g. in squamous cell carcinoma of lung [64], and uterine cervix cancer [65]. The diverse role of PDPN in various human cancers was also highlighted by the recent study of Tsuneki *et al*., 2013 [66] which demonstrated that the primary function of podoplanin in oral squamous cell carcinoma is cell adhesion to the ECM, with no effect on cell migration. The apparently contradictory PDPN activities suggest that podoplanin might function as a factor promoting or suppressing cancer metastatic potential and progression depending on the tissue- and organ-specific environment, and on the cellular context.

Podoplanin is found in the membrane lipid rafts and interacts with other raft- specific proteins, like CD44, a marker of epithelial-mesenchymal transition, as well as ezrin and moesin, the cytoskeletal organizer proteins from ERM family [6 1, 67]. CD 44 is implicated in the tumor growth and progression and can act as a co-receptor modulating signal transduction through cell surface tyrosine kinase receptors, and this function depends upon its interaction with ERM proteins [68]. Binding of podoplanin to these proteins through its cytoplasmic domain anchors PDPN to the actin cytoskeleton, what leads to the activation of small Rho GTPases and the induction of cell migration and invasion, as well as epithelilal-mesenchymal transition [21], [56], [69]. Moreover, as demonstrated recently, intracellular domain of podoplanin undergoes cleavage by γ-secretase, releasing a short intracellular domain into cytosol, which is suggested to play an important role in the podoplanin signaling and function [70]. Furthermore, a number of other proteins have been proposed to act together with podoplanin in controlling cell motility, migration and invasion, including molecules of the extracellular matrix [21], [56], [71], and matrix metalloproteinases (MMP-1, MMP-2, MMP-9, MMP-10), which are also thought to be involved in PDPN-dependent tumor progression [72], [73]. Thyroid tumor signaling pathways involve a broad variety of secondary molecular alterations and up-regulation of variety of proteins involved in tumor progression [2], [74], [75]. Many of them are non-cellular components of extracellular matrix, which, in human tumors, undergoes composition and organization remodeling what might influence adhesion, migration, invasiveness and other functional properties of tumor cells.

In summary, this is the first evaluation of the expression and function of podoplanin in thyroid cancer, and our results strongly suggest that the malignant potential of PTCs may be related to PDPN expression. Our findings and other available data indicate that the uncharacterized mechanisms by which PDPN affects the aggressive phenotype of cancer cells are complex and may be dependent on the examined tumor tissue. The molecular mechanisms by which PDPN can affect the metastatic phenotype of the thyroid cancer cells have yet to be revealed. Further studies are necessary to elucidate the detailed mechanism/s by which PDPN expression is regulated in differentiated thyroid carcinoma cells and contributes to metastatic potential of thyroid tumors.
